# Dual-Template Molecularly Imprinted Polymers for Dispersive Solid-Phase Extraction Combined with High Performance Liquid Chromatography for the Determination of Sulfonamide Antibiotics in Environmental Water Samples

**DOI:** 10.3390/polym16213095

**Published:** 2024-11-01

**Authors:** Yuhao Wen, Mingyang Hou, Xingkai Hao, Dani Sun, Hao Zhang, Farooq Saqib, Wenhui Lu, Huitao Liu, Lingxin Chen, Jinhua Li

**Affiliations:** 1 Coastal Zone Ecological Environment Monitoring Technology and Equipment Shandong Engineering Research Center, Shandong Key Laboratory of Coastal Environmental Processes, Shandong Research Center for Coastal Environmental Engineering and Technology, Yantai Institute of Coastal Zone Research, Chinese Academy of Sciences, Yantai 264003, China; wenyuhao115@163.com (Y.W.); 19508631747@163.com (M.H.); sun19961212@163.com (D.S.); zhanghao798940@163.com (H.Z.); lxchen@yic.ac.cn (L.C.); 2 College of Chemistry and Chemical Engineering, Yantai University, Yantai 264005, China; 3 ZJU-Hangzhou Global Scientific and Technological Innovation Center, Zhejiang University, Hangzhou 311215, China; haoxingkai@hotmail.com (X.H.); 0623099@zju.edu.cn (F.S.); 4 Faculty of Light Industry, Qilu University of Technology (Shandong Academy of Sciences), Jinan 250353, China; whlu@qlu.edu.cn

**Keywords:** molecularly imprinted polymers, dual-template imprinting, sulfonamide antibiotics, dispersive solid-phase extraction, environmental water

## Abstract

In this study, we designed a molecularly imprinted polymers-dispersive solid-phase extraction-high-performance liquid chromatography (MIPs-DSPE-HPLC) method, as a simple and efficient platform for the sensitive detection of two sulfonamide antibiotics (SAs) of sulfamethoxine (SMM) and sulfamethoxazole (SMZ) in environmental water samples. Using SMM and SMZ as templates, methacrylic acid as the functional monomer, ethylene glycol dimethacrylate as the crosslinking agent, and azodiisobutyronitrile as the catalyst, the dual-template molecularly imprinted polymers (dt-MIPs) were successfully synthesized via surface imprinting technology and multi-template imprinting strategy. The adsorption properties of the prepared MIPs were characterized, and the adsorption capacities of MIPs towards SMZ and SMM were 27.35 mg/g and 30.92 mg/g, respectively. The detection limits of the method in three environmental water samples were in the range of 0.23–1.74 μg/L, and the recoveries were between 82.7 and 110.3%, with relative standard deviations less than 5.93%. The construction process of this MIPs-DSPE-HPLC method is straightforward, exhibits high sensitivity and selectivity, and thus provides a versatile method for the quantification of SAs in complex matrices.

## 1. Introduction

Sulfonamide antibiotics (SAs) are a class of synthetic broad-spectrum antibiotics characterized by the presence of an aminobenzenesulfonamide structure [1,2]. They are widely used in various fields, including healthcare, veterinary medicine, and aquaculture, due to their efficacy, low cost, and availability. However, the excessive use of SAs can lead to the proliferation of resistant bacteria, resulting in reduced therapeutic effectiveness [3]. Additionally, SAs may cause adverse effects in humans, including renal damage, gastrointestinal disturbances, and allergic reactions, and there is also a potential risk of carcinogenicity. Furthermore, SAs can disrupt microbial communities in the environment; their overuse in aquaculture and livestock farming may result in the accumulation of unmetabolized antibiotics in animal-derived food products, such as milk, eggs, and pork [4]. Consequently, there is a pressing need to develop standardized analytical methods for monitoring and assessing the levels of SAs in complex matrices.

At present, commonly used methods for the detection of SAs include high performance liquid chromatography (HPLC) [5], enzyme-linked immunosorbent assay [6], spectroscopy [7], and electrochemistry [8,9]. Among these, HPLC is a widely employed analytical technique for the separation and determination of compounds, demonstrating significant efficacy in discriminating among various components within complex mixtures. Robust performances make it an essential tool in both research and industrial applications for compound analysis [10]. Furthermore, this methodology is capable of detecting target substances at remarkably low concentrations, making it particularly suitable for trace analysis. As one of the most widely used separation and analysis methods, HPLC is now widely used for the detection of antibiotics in a variety of complex matrices. For example, Pamreddy et al. [5] used a hydrophilic–lipophilic balance column to extract SAs and TCs from sewage sludge and detected them by HPLC tandem mass spectrometry (HPLC-MS/MS), in which the recovery rates of SAs were 90.4–99.9% and the limits of detection (LODs) were 0.6–4.2 μg/kg.

It is essential to employ sample pretreatment techniques that quantitatively transfer the target components into a solution for subsequent analysis [11], to enhance the performance and accuracy of analytical detection methods and protect instruments. Dispersive solid-phase extraction (DSPE) is a commonly utilized sample pretreatment technique based on the principles of liquid–solid chromatography, utilizing selective adsorption and selective elution to enrich, separate, and purify targets. However, in complex sample matrices, a variety of interfering substances may be present. These interferences can adversely affect the recovery and purity of the target analytes. To address this challenge, functional materials have been widely adopted [12,13,14]. For example, the use of molecularly imprinted polymers (MIPs) as adsorbents for DSPE can improve the selectivity and specificity of traditional DSPE [15,16]. Samples pretreated with molecular imprinting-based DSPE (MI-DSPE) can improve the analytical performance of various detection methods, and have been increasingly used in antibiotic determination.

In this study, we developed a molecularly imprinted polymers-dispersive solid-phase extraction-HPLC (MIPs-DSPE-HPLC) method for the simultaneous enrichment, separation, and detection of sulfamethoxazole (SMZ) and sulfamethoxine (SMM). The results indicated that the synthesized MIPs exhibited excellent adsorption capacities for SMZ (27.35 mg g⁻^1^) and SMM (30.92 mg g⁻^1^). Furthermore, the method was applied to three different environmental water samples, yielding LOD (0.23–1.74 μg/L), good recovery rates (82.7–110.3%), and acceptable accuracy (relative standard deviations (RSDs) less than 5.93%). The proposed MIPs-DSPE-HPLC method demonstrated significant advantages in selectivity, sensitivity, and accuracy, establishing it as a versatile solution for monitoring trace levels of SAs in complex matrices.

## 2. Materials and Methods

### 2.1. Reagents and Materials

Methanol (CH_3_OH), ethanol (C_2_H_5_OH), acetic acid (CH_3_COOH), acetonitrile (CH_3_CN), sodium dihydrogen phosphate (NaH_2_PO_4_), methylbenzene, sodium hydroxide (NaOH), and sodium tetraborate decahydrate (Na_2_B_4_O_7_·10H₂O) were purchased from Sinopharm Chemical Reagent Co., Ltd. (Shanghai, China). Tetraethyl orthosilicate (TEOS), ethylene glycol dimethacrylate (EGDMA), γ-(methacryloyloxy) propyltrimethoxysilane (MPS), azodiisobutyronitrile (AIBN), ammonia (NH_3_·H_2_O), and methacrylic acid (MAA) were purchased from Shanghai Aladdin Biochemical Technology Co., Ltd. (Shanghai, China). Norfloxacin (NOR), sulfamine, SMZ, Sulfadiazine (SDZ), Sulfapyridine, and SMM were purchased from Shanghai McLean Biochemical Technology Co., Ltd. (Shanghai, China). Hydrochloric acid (HCl) was purchased from Tianjin Comio Chemical Reagent Co., Ltd. (Tianjin, China). All the chemical reagents used in this study were of analytical pure grade, which had a purity greater than 99%, except CH_3_OH and CH_3_CN, which had a chromatographic pure grade of over 99.7%. Ultrapure water of 18.2 MΩ specific resistance (Millipore, Bedford, MA, USA) was used throughout all experiments.

### 2.2. Instrumentation

Optimized Sas’ separation was achieved in a phase column (Aces aq C18, 250 × 4.6 mm i.d., particle size: 5 µm) by isocratic elution using a mobile phase consisting of CH_3_OH–water (containing 0.1% formic acid) at a flow rate of 0.3 mL/min. The injection volume, column temperature, and detection wavelength were individually set as 20 µL, 25 °C, and 278 nm.

The morphologies of the polymer particles were examined by scanning electron microscopy (SEM, Hitachi S-4800, Tokyo, Japan, 3 kV, 20 μA). All samples were sputter-coated with thin gold film before observation. The infrared spectra of the samples were obtained using a Fourier transform infrared (FT-IR) spectrometer (Shimadzu Spectrum 100 N, Kyoto, Japan, 400–4000 cm^−1^, resolution: 16) by using a pressed KBr tablet.

### 2.3. General Approach

**Figure 1:**
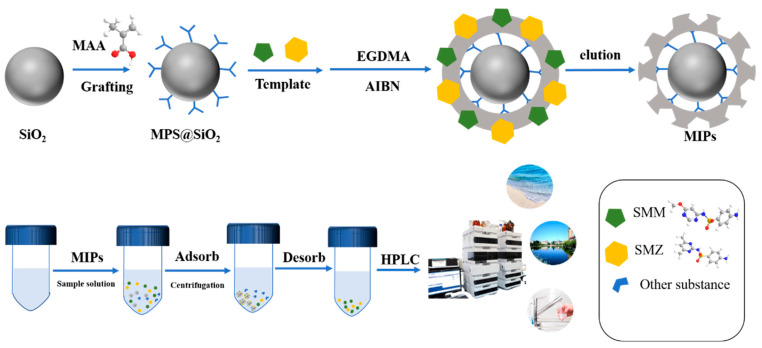
Schematic diagrams of the dt-MIPs’ preparation (**up**) and DSPE-HPLC process (**below**).

To prepare MIPs for the recognition and adsorption of template molecules, SiO_2_ was utilized as the support material, with SMM and SMZ serving as template molecules, and MAA was employed as the functional monomer, MPS as the surfactant, EGDMA as the crosslinking agent, and AIBN as the initiator. Through surface imprinting technology and a multi-template imprinting strategy, we synthesized the polymers. Subsequently, the template molecules were removed using organic solvents, resulting in MIPs@MPS@SiO_2_, namely, the dual-template MIPs (dt-MIPs) or MIPs for simplicity, which possess specific recognition sites towards template molecules. Consequently, the MIPs were employed for the preprocessing of samples to selectively enrich the antibiotics present in the samples. The enriched samples were then subjected to HPLC analysis, allowing for the quantification of the target substances, thereby successfully establishing the MIPs-DSPE-HPLC method. Figure 1 schematically shows the preparation and application of MIPs.

### 2.4. Preparation of SiO_2_

To synthesize the SiO_2_ nanoparticles, a well-established method was employed with minor modifications. Specifically, 50 mL of C_2_H_5_OH and 4 mL of NH_3_·H_2_O were added to a 100 mL round-bottom flask. After homogenization using magnetic stirring, a mixture consisting of 7 mL of C_2_H_5_OH and 3 mL of TEOS was introduced into a dropping funnel, with the flow rate controlled between 3 and 5 s per drop. The reaction was vigorously stirred at room temperature for a duration of 12 h. Upon completion of the reaction, the mixture was centrifuged at 8000 rpm for 5 min to separate the supernatant. The resulting products were washed three times with C_2_H_5_OH, ensuring the complete dispersion of SiO_2_ during each wash before centrifugation. Subsequently, the washing procedure was repeated three times using water, followed by vacuum drying for 8 h. The obtained solid was stored under 4 °C for subsequent experiments.

### 2.5. Preparation of MPS@SiO_2_

To facilitate the coating of MPS onto the surface of SiO_2_, a well-established method was employed. Specifically, 1 g of SiO_2_ was placed into a round-bottom flask. Subsequently, 50 mL of toluene was added, and ultrasonication was utilized to ensure the uniform dispersion of SiO_2_ in the toluene. Following this, 2.5 mL of MPS was introduced, and the mixture was allowed to react at 90 °C for 24 h. After the reaction, the mixture was cooled to room temperature and centrifuged at 8000 rpm for 5 min to separate the solid. The resulting product was washed three times with C_2_H_5_OH and then subjected to vacuum drying for 6 h. The obtained solid was stored under 4 °C for subsequent experiments. Note that the reaction system should be flushed by N_2_ for at least half an hour to remove O_2_ before the reaction starts.

### 2.6. Preparation of MIPs@MPS@SiO_2_

To prepare the MIPs on the surface of MPS@SiO_2_, 1 mM of SMM and 1 mM of SMZ were added to a round-bottom flask, followed by the addition of 10 mL of CH_3_CN and 70 μL of MAA. The prepolymerization was conducted at 4 °C for 12 h. Subsequently, 0.8 mL of EGDMA was introduced into the mixture. A total of 100 mg of MPS@SiO_2_ was added in a beaker, to which 15 mL of CH_3_CN was added. Subsequently, the obtained solution was added to the prepolymerization system, and 0.12 g of AIBN was added. The mixture was stirred at a low speed and allowed to react at 60 °C for 24 h. After the reaction, the product was allowed to cool naturally to room temperature and was then separated by centrifugation. The resulting material was washed three times with a CH_3_OH: CH_3_COOH (*v*:*v* = 9:1) and subjected to vacuum drying for 12 h. Note that the reaction system should be flushed by N_2_ for at least 45 min to remove O_2_ after AIBN was added. Additionally, to evaluate the specific adsorption performance of the MIPs, a control group of non-imprinted polymers (NIPs) was established, differing in that the template molecules SMM and SMZ were not included during the preparation process.

### 2.7. Adsorption Experiments

#### 2.7.1. Static Adsorption Test

To evaluate the adsorption performance of the MIPs, both static and dynamic adsorption experiments were conducted, and the adsorption capacity of the MIPs was calculated. In the static adsorption experiment, 10 mg of MIPs was dispersed in 8 mL of standard solutions of SMM or SMZ at concentrations ranging from 5 to 100 mg/L. The mixtures were agitated at room temperature for 6 h to facilitate adsorption. Approximately 1 mL of the solution was then collected, filtered through a membrane, and analyzed using HPLC to determine the Q value of the MIPs. For the dynamic adsorption experiment, 10 mg of MIPs was dispersed in 8 mL of standard solutions of SMM or SMZ at a concentration of 100 mg/L. The adsorption time varied from 5 to 100 min. At each time point, approximately 1 mL of the sample was taken, filtered through a membrane, and analyzed by HPLC to calculate the Q value of the MIPs at different time intervals. Additionally, NIPs were established as a control group for the experiments mentioned above, and the Q values for the NIPs were calculated to further assess the adsorption performance of the MIPs.

#### 2.7.2. Adsorption Selectivity Test

To evaluate the recognition capability and affinity of the MIPs for structurally diverse molecules, we selected SDZ, which has a similar structure to the two template molecules under investigation. Additionally, NOR, a molecule with a significantly different structure from the template molecules, was introduced to further validate the selective advantages of the MIPs prepared using a dual-template imprinting strategy. A total of 10 mg of MIPs@MPS@SiO_2_ and NIPs@MPS@SiO_2_ was separately dispersed in 8 mL of standard solutions containing 100 mg/L of SDZ and NOR, respectively. The mixtures were agitated for 6 h to reach adsorption equilibrium. Following this, the supernatant was collected via centrifugation and filtered through a 0.22 µm microporous filter membrane. The concentration of the analytes was then determined using HPLC, and the Q value was calculated.

## 3. Results and Discussion

### 3.1. Characterization of SiO_2_, MIPs, and NIPs

**Figure 2:**
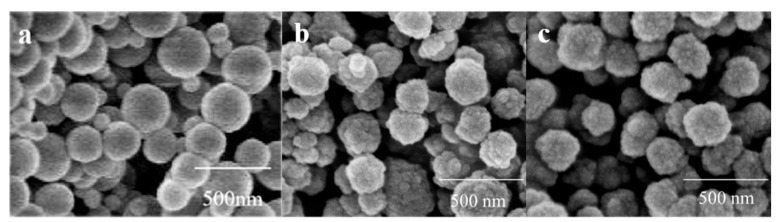
SEM images of (**a**) SiO_2_, (**b**) MIPs, and (**c**) NIPs.

To determine whether the synthesized MIPs possess specific recognition sites on their surface, we examined the surface morphology of SiO_2_, MIPs, and NIPs using SEM (Figure 2). In comparison to SiO_2_, both MIPs and NIPs exhibit a rougher spherical morphology; however, MIPs display a greater degree of surface roughness than NIPs. This phenomenon can be attributed to the incorporation of two distinct template molecules within the MIPs. The functional monomers undergo pre-assembly and polymerization reactions with the different template molecules, resulting in the formation of polymer microspheres with uneven sizes and shapes. This observation further confirms the presence of specific recognition sites on the surface of MIPs, whereas NIPs lack template molecules, leading to uniformly distributed binding sites and a relatively smoother surface compared to MIPs. These results provide compelling evidence for the successful preparation of MIPs.

**Figure 3:**
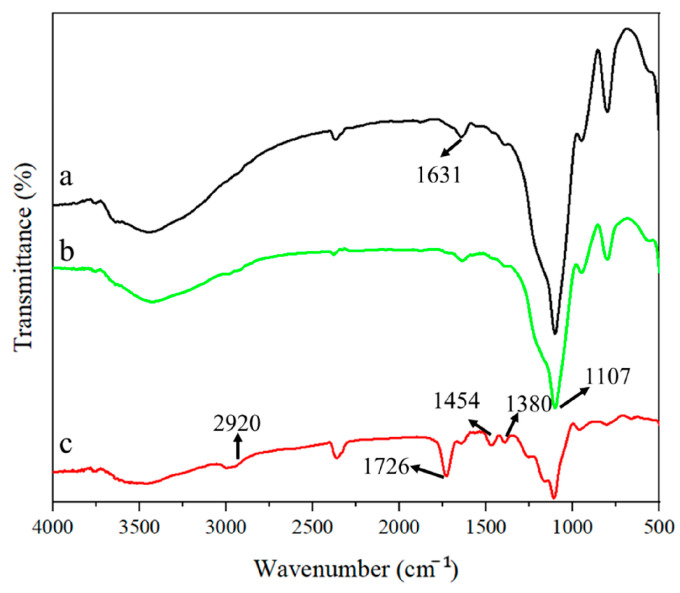
FT-IR spectra of nanoparticles (**a**) MPS@SiO_2_; (**b**) SiO_2_; and (**c**) MIPs@MPS@SiO_2_.

To further confirm the successful encapsulation of MIPs and NIPs, we employed FT-IR spectroscopy to characterize the functional groups present in the synthesized materials. As illustrated in Figure 3, the curve labeled a exhibits a characteristic peak at 1631 cm^−1^ corresponding to the C=C stretching vibration, while a peak at 1107 cm^−1^ is attributed to SiO_2_. The curve labeled c encompasses the characteristic peaks of SiO_2_ and C=C, along with additional peaks at 1726 cm^−1^ and 1380 cm^−1^, which are associated with the C=O vibrational mode and C-H stretching vibrations of EGDMA, respectively. Furthermore, peaks at 2920 cm^−1^ and 1454 cm^−1^ correspond to the O-H stretching vibration and the –COO- stretching vibrations of MAA. These findings indicate the successful incorporation of MAA and EGDMA in the polymer matrix, thereby confirming the successful preparation of MIPs.

### 3.2. Adsorption Performance

**Figure 4:**
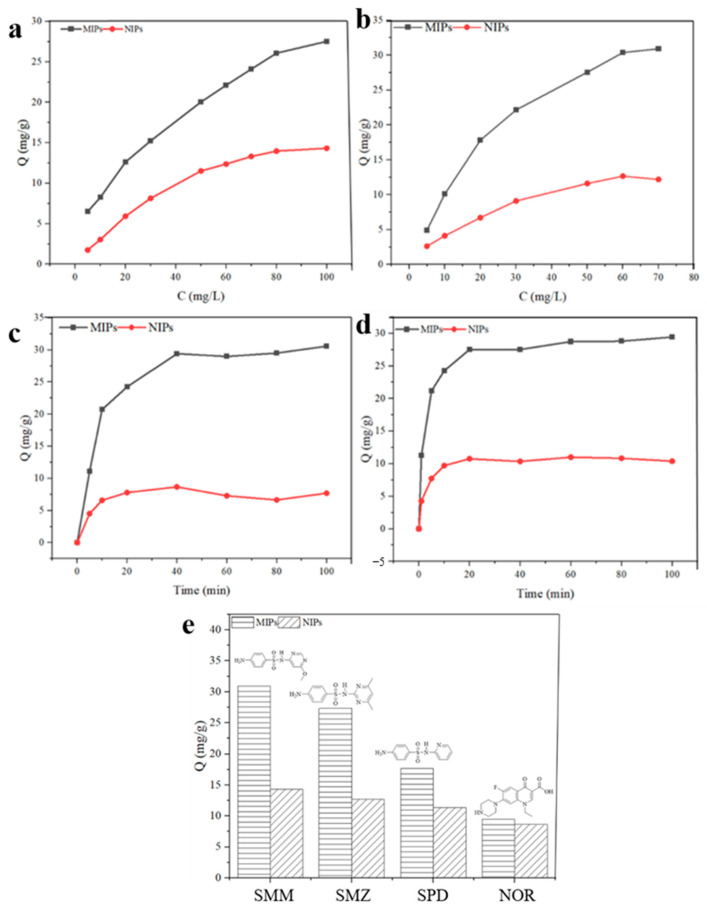
Adsorption performance: static adsorption curves of (**a**) SMZ and (**b**) SMM; dynamic adsorption curves of (**c**) SMZ and (**d**) SMM; and (**e**) selectivity of MIPs and NIPs for the four SAs.

To evaluate the adsorption performance of MIPs, we conducted both static and dynamic adsorption experiments and calculated the adsorption capacity of the MIPs. The static adsorption isotherms are illustrated in Figure 4a,b. Within the concentration range from 5 to 80 mg/L for SMZ, the adsorption amount of MIPs increased with the rising concentration. In the 80 to 100 mg/L range, the increase in adsorption was minimal, with the maximum adsorption capacity for SMZ recorded at 27.53 mg/g (Figure 4a). For SMM, within the concentration range from 5 to 60 mg/L, the adsorption amount also exhibited an increase with rising concentration; however, after 60 to 70 mg/L, the increase became negligible (Figure 4b). Notably, due to the absence of specific adsorption sites on their surface, NIPs showed significantly reduced adsorption capacities for both SMM and SMZ.

Furthermore, the dynamic adsorption profiles are shown in Figure 4c,d. During the initial stage (0 to 10 min), both MIPs and NIPs exhibited a rapid increase in adsorption amounts. After 20 min, the adsorption amount of NIPs for SMZ showed little to no variation, while MIPs continued to adsorb SMZ, with an increase observed between 20 and 40 min, ultimately reaching adsorption equilibrium by the 40 min mark (Figure 4c). In contrast, the adsorption of NIPs for SMM remained relatively constant after 10 min, whereas MIPs achieved equilibrium at the 20 min mark (Figure 4d). SMZ and SMM reached adsorption equilibrium at 40 and 20 min, respectively, and this indicates the presence of the second-stage internal adsorption within the imprinted layer of the dual-template MIPs [17,18].

To further validate the high selectivity of MIPs for template molecules, we conducted adsorption experiments using structural analogs of the template molecule, SMZ, specifically SDZ, alongside a non-analog, NOR. The adsorption capacities of the MIPs and NIPs for these substances are presented in Figure 4e. MIPs demonstrated a significantly higher adsorption capacity for the template molecules SMZ and SMM, with values of 27.35 mg/g and 30.92 mg/g, respectively. In contrast, NIPs exhibited markedly lower adsorption capacities for these two substances, measuring 14.3 mg/g and 12.7 mg/g, respectively. When examining the adsorption performance of MIPs towards the structural analog SDZ, a slight decrease in adsorption capacity was observed. Conversely, MIPs displayed the weakest recognition performance for NOR, a compound with a significantly different structure from the template molecules. This finding further substantiates the specific selectivity of MIPs towards particular template molecules.

### 3.3. Adsorption Data Model Fitting

To conduct a comprehensive analysis of the isothermal adsorption characteristics of the materials, two widely accepted thermodynamic models of Langmuir and Freundlich were employed for evaluation [19,20,21]. The results of the static data fitting are illustrated in Figure S1 and presented in Table S1. Using the Langmuir adsorption model, the maximum adsorption capacities for MIPs were found to be 37.4 mg/g for SMZ and 40.4 mg/g for SMM. The actual adsorption quantities obtained through the Freundlich isotherm model were 27.5 mg/g for SMZ and 12.7 mg/g for SMM. Moreover, we established NIPs as a control group, evaluating both adsorption models for comparative analysis. The results indicate that the maximum adsorption capacities for NIPs, assessed using the Langmuir model, were significantly lower for SMZ (18.5 mg/g) and SMM (19.7 mg/g) compared to those of MIPs. Correspondingly, findings from the Freundlich model also demonstrated lower values for NIPs (SMZ: 14.3 mg/g; SMM: 12.7 mg/g). These results confirm that the dual-template MIPs possess a greater number of adsorption sites and exhibit higher adsorption capacities for both template molecules. Analyzing the adsorption fitting outcomes suggests that MIPs demonstrate good fitting correlations with both models, indicating the presence of uniform monolayer adsorption as well as internal imprinting layer recognition. In contrast, NIPs showed a superior fitting correlation with the Langmuir isotherm model, with a correlation coefficient exceeding 0.99, while presenting a lower correlation coefficient for the Freundlich isotherm model, suggesting that NIPs primarily engage in surface monolayer adsorption without specific adsorption characteristics.

To investigate the dynamic adsorption processes of MIPs and their key parameters, this study utilized three classical kinetic models: pseudo-first-order kinetics, pseudo-second-order kinetics, and intraparticle diffusion models [20,21]. The kinetic data obtained for SMZ and SMM were analyzed using the pseudo-first-order kinetic equation and the pseudo-second-order kinetic rate equation, with the results presented in Figure S2 and Table S2. The findings indicate that MIPs exhibited the best fit for the pseudo-second-order kinetic model regarding SMZ, with a linear correlation coefficient of 0.989. The theoretical adsorption capacity calculated from the equation was 32.9 mg/g, which closely aligned with the actual maximum dynamic adsorption of 30.8 mg/g. Similarly, for SMM, the pseudo-second-order kinetic model provided a superior fit, yielding a linear correlation coefficient of 0.995; the theoretical adsorption capacity was calculated to be 27.8 mg/g, while the actual adsorption was determined to be 29.4 mg/g. Based on the assumptions made regarding the pseudo-second-order kinetic model, the adsorption of both types of SAs by MIPs can be classified as chemical adsorption. Intraparticle diffusion results are illustrated in Figure S3, revealing that the adsorption process of MIPs consists of two distinct stages. The initial adsorption rates K_id_ for SMZ and SMM were 10.36 and 6.11, respectively, indicating rapid adsorption with a relatively thin boundary layer. As surface adsorption sites become occupied by the target molecules, the diffusion of template molecules into the inner layers of the material slows due to spatial hindrance and other factors, resulting in a decrease in the adsorption rate, as evidenced by a marked reduction in the slope of the equation to 2.06 for SMZ and 1 for SMM. In contrast, NIPs reached equilibrium after only one stage of adsorption.

### 3.4. Optimization of MIPs-DSPE Conditions

To investigate the optimal performance of the developed MIPs-DSPE-HPLC method, we optimized various parameters affecting the adsorption properties of the MIPs. These parameters included the amount of MIPs used, the pH of the extraction solution, extraction time, the volume of the elution solvent, and desorption time.

**Figure 5:**
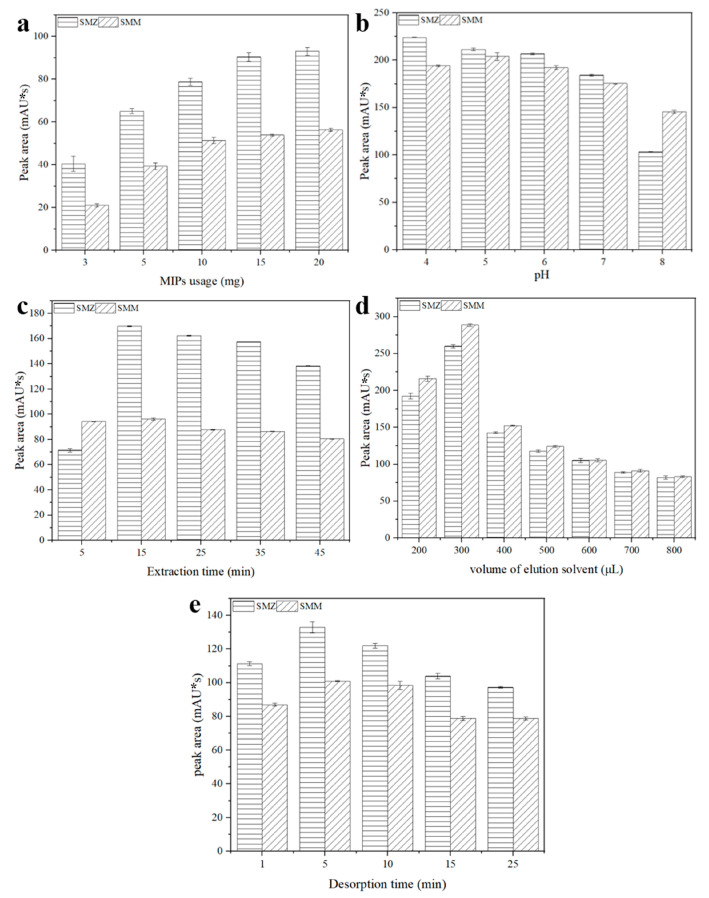
Effects of different conditions for extraction efficiency of DSPE: (**a**) MIPs’ dosage, (**b**) sample pH, (**c**) extraction time, (**d**) volume of elution solvent, and (**e**) desorption time.

To achieve maximum extraction efficiency, we varied the amount of MIPs used in the experiments, setting it at 3, 5, 10, 15, and 20 mg, and measured the amount of target analytes desorbed accordingly. As depicted in Figure 5a, within the range of 3 to 20 mg of MIPs, the desorption area for SMZ increased with the escalating amount of MIPs, reaching its peak at 20 mg, which was slightly higher than that observed at 15 mg. Conversely, the peak area for SMM exhibited minimal variation when the amount of MIPs was set at 10, 15, and 20 mg. Taking these results into account, we selected an MIP quantity of 15 mg for subsequent experiments.

To identify the optimal pH value of the extraction solution for maximizing the extraction efficiency of MIPs, we conducted an optimization study on the pH of SMM and SMZ solutions, with the results presented in Figure 5b. Under acidic conditions, the pH of the extraction solution had a negligible effect on extraction efficiency. However, when the pH exceeded 7, a significant reduction in the extraction efficiency of MIPs was observed. At a pH range from 5 to 7, SMM and SMZ predominantly exist in their non-ionized forms, allowing the recognition between MIPs and the target analytes to primarily rely on hydrogen bonding, which aligns with the recognition sites, resulting in higher adsorption capacities. Therefore, considering these findings, a pH value of 5 was selected for subsequent experiments.

To determine the optimal extraction time for achieving maximum extraction efficiency, we measured the adsorption capacity of MIPs within a time range of 5 to 45 min, and calculated the extraction efficiency, as shown in Figure 5c. At an extraction time of 5 min, the extraction efficiency of MIPs for SMZ was significantly lower compared to that at 15 min, while the extraction efficiency for SMM did not differ substantially from that at 15 min. As the extraction time was extended beyond 15 min, a slight decrease in extraction efficiency was observed. The dynamic adsorption results indicated that SMM reached equilibrium more rapidly than SMZ, which may be attributed to the greater steric hindrance of SMZ, resulting in a longer internal adsorption time. Therefore, considering these findings, an extraction time of 15 min was selected for subsequent experiments.

To determine the optimal volume of the desorption solvent for maximizing extraction efficiency, we utilized a mixed solution of CH_3_CN and CH_3_COOH (*v*:*v* = 9:1) as the desorption solvent and optimized its volume within the range of 200 to 800 μL, with the results presented in Figure 5d. At a desorption solvent volume of 200 μL, desorption was incomplete, leading to low extraction efficiency. Conversely, the extraction efficiency peaked at 300 μL. However, when the volume of the desorption solvent exceeded 400 μL, a marked decrease in extraction efficiency was observed. This decline can be attributed to the dilution of the analytes’ concentrations and the decrease in enrichment efficiency, resulting from the addition of excess desorption solvent after complete extraction [22,23]. Therefore, a desorption solvent volume of 300 μL was selected for subsequent experiments.

To ascertain the optimal desorption time for maximizing extraction efficiency, we investigated the impact of desorption duration on extraction efficiency within the range from 1 to 25 min, with the results illustrated in Figure 5e. At a desorption time of 5 min, the extraction efficiency was at its highest; however, at a desorption time of 1 min, desorption was incomplete, resulting in a lower peak area. Beyond a desorption time of 10 min, a decline in extraction efficiency was observed, which may be attributed to the re-adsorption of desorbed SMZ and SMM onto the MIPs. Therefore, considering these findings, a desorption time of 5 min was selected for subsequent experiments.

### 3.5. Method Validation of the dt-MIPs-DSPE

To validate the established MIPs-SPE-HPLC method, we performed enrichment and separation detection of SMM and SMZ in standard solutions under optimal conditions. The investigation included parameters such as linearity, linear range, LOD (Limit of Detection), and LOQ (Limit of Quantification), with the results summarized in Table 1. The linear ranges for SMZ and SMM were found to be 0.01 to 5 μg/L, with correlation coefficients exceeding 0.999. The LODs, calculated based on a signal-to-noise ratio (S/N) of 3, were 0.23 and 0.27 μg/L, respectively. The LOQs, determined from an S/N of 10, were 0.72 and 0.83 μg/L, respectively. The established MIPs-DSPE-HPLC method demonstrated a wide linear range and high sensitivity. Furthermore, using a mixed solution of both SAs at a concentration of 100 μg/L, we assessed the intraday and interday precision by conducting five parallel measurements. The intraday precision, represented by the relative standard deviation (RSD, *n* = 5), was recorded at 0.14% to 0.35% for SMZ and SMM, while the interday precision ranged from 0.54% to 1.12%. These findings indicate that the developed MIPs-DSPE-HPLC method exhibits stable performance and is capable of simultaneously quantifying the two SAs in environmental water samples.

### 3.6. Practical Application of dt-MIPs-DSPE-HPLC

To validate the analytical performance of the established MIPs-DSPE-HPLC method for real sample analysis, we employed it to detect three types of water samples: seawater, lake water, and tap water. We generated calibration curves and examined the linear relationships, LOD and LOQ, with the results summarized in Table 2. The developed method demonstrated correlation coefficients greater than 0.991 for both SMZ and SMM across the three environmental water samples, with a linear range of 5 to 500 μg/L. The LODs ranged from 0.23 to 1.74 μg/L, and the LOQs ranged from 0.77 to 5.32 μg/L. Furthermore, the MIPs-DSPE-HPLC method exhibited a wide linear range and high sensitivity. Therefore, the established MIPs-DSPE-HPLC method demonstrates commendable practicality for analysis in real samples.

**Figure 6:**
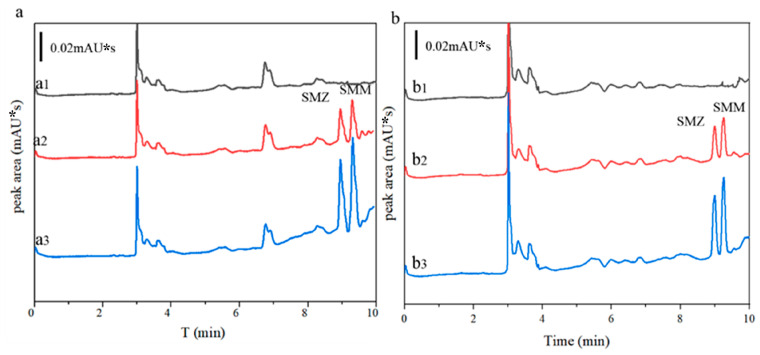
HPLC chromatogram of sample: (**a**) seawater sample, (a1) blank sample, (a2) spiked with 30 μg/L, (a3) spiked with 100 μg/L; (**b**) tap water sample, (b1) blank sample, (b2) spiked with 30 μg/L, (b3) spiked with 100 μg/L.

Additionally, we conducted recovery experiments by spiking two concentrations (30 and 100 μg/L) in the three types of environmental water samples, with the results presented in Table 3. The chromatograms for the spiked seawater and tap water are illustrated in Figure 6. The recovery rates for lake water, seawater, and tap water were found to be 96.3% to 106.2%, 82.7% to 110.3%, and 92.2% to 107.3%, respectively, with RSDs of 3.82% to 8.24%, 1.27% to 5.93%, and 1.78% to 4.89%. Therefore, the established method exhibits reliable recovery rates, demonstrating its suitability for the determination of trace SAs in environmental water samples.

### 3.7. Method Performance Comparison

To further assess the performance of the MIPs-DSPE-HPLC method, the developed method was compared with other relevant detection methods for SAs, as shown in Table S1 [24,25,26,27]. In comparison to other methods, the approach developed in this study demonstrates a lower LOD. Additionally, the use of the DSPE method for sample pretreatment enhances the efficiency of the preprocessing stage. The core-shell structured MIPs synthesized in this experiment achieve adsorption equilibrium in a shorter time frame compared to alternative methods. Collectively, these findings indicate that the method established in this research exhibits superior performance.

## 4. Conclusions

This study focuses on two types of SAs and employs MIPs for the specific binding in DSPE coupled with chromatographic detection. A dt-MIPs-DSPE-HPLC method has been developed, which demonstrates high selectivity and sensitivity, providing a universal approach for the rapid adsorption and detection of target analytes in complex matrices. Furthermore, this method has potential for further optimization. Currently, it faces challenges such as a cumbersome operation and prolonged detection time. Future work may involve optimizing the sample preparation techniques and developing corresponding portable devices to enhance the method’s portability and universality and broaden its application scope. And instead of only the results of research work in the lab, a large-scale transfer to industrial and agricultural practices of related materials, methods, and technologies should be intensively carried out.

## Figures and Tables

**Table 1:** Analytical performance of dt-MIPs-DSPE-HPLC in standard solutions.

SAs	Curve	*R* ^2^	Linear Range (μg/L)	LOD (μg/L)	LOQ (μg/L)
SMZ	y = 31,724x + 763.27	0.9993	1–500	0.23	0.72
SMM	y = 293,721x + 283.3	0.9995	1–500	0.27	0.83

**Table 2:** Analytical performance of dt-MIPs-DSPE-HPLC method in actual samples.

Samples	SAs	Curve	*R* ^2^	Linear Range (μg/L)	LOD (μg/L)	LOQ (μg/L)
Tap water	SMZ	y = 29,048x + 1745.4	0.9995	5–500	0.23	0.77
	SMM	y = 24,558x + 640.54	1.0000	5–500	0.34	1.09
Seawater	SMZ	y = 47,148x − 430.11	0.9996	5–500	0.43	1.59
	SMM	y = 42,841x + 365.58	0.9998	5–500	0.56	1.96
Lake Water	SMZ	y = 13,172x + 2087	0.9991	10–500	1.08	3.32
	SMM	y = 13,172x + 2087	0.9991	10–500	1.74	5.32

**Table 3:** Recoveries of spiked SAs in real samples by dt-MIPs-DSPE-HPLC.

SAs	Spiked Concentration (μg/L)	Lake Water		Seawater		Tap Water	
		Recovery (%)	RSD (%)	Recovery (%)	RSD (%)	Recovery (%)	RSD (%)
SMZ	0	0	-	0	-	0	-
	30	106.2	4.2	82.7	5.93	107.3	4.89
	100	99.7	6.60	92.9	1.27	95.1	1.78
SMM	0	0	-	0	-	0	-
	30	96.3	8.24	110.3	4.97	92.2	1.85
	100	102.4	3.82	102.8	4.29	106.1	3.6

## Data Availability

Data will be made available on request.

## Supplementary Materials

The following supporting information can be downloaded at: https://www.mdpi.com/article/10.3390/polym16213095/s1, Figure S1: Langmuir adsorption curve fitting of (a) SMZ and (b) SMM; Freundlich adsorption curve fitting of (c) SMZ and (d) SMM; Figure S2: (a) and (b) fitting the SMZ pseudo first and pseudo second order kinetic adsorption curves; (c) and (d) fitting the SMM pseudo first and pseudo second order kinetic adsorption curves; Figure S3: Internal adsorption curves of (a) SMZ and (b) SMM; Table S1: Langmuir and Freundlich adsorption fitting data of dt-MIPs and NIPs; Table S2: Three kinetic models fitting of dt-MIPs and NIPs; Table S3: Analytical performance comparison of the dt-MIPs-DSPE-HPLC with reported SPE based methods for SAs determination. References [19,20,21,24,25,26,27] have been cited in the Supplementary Materials.
