# Supplementary material for: Dual-Template Molecularly Imprinted Polymers for Dispersive Solid-Phase Extraction Combined with High Performance Liquid Chromatography for the Determination of Sulfonamide Antibiotics in Environmental Water Samples
Source: Polymers (Basel). 2024 Nov 1;16(21):3095. doi: 10.3390/polym16213095 (PMC11548663; doi:10.3390/polym16213095)
Supplement: Supplementary file 1 [file polymers-16-03095-s001.zip › polymers-3268297-supplementary.pdf]

Supporting Information

Dual-Template Molecularly Imprinted Polymers for Dispersive Solid-Phase Extraction Combined with High Performance Liquid Chromatography for the Determination of Sulfonamide Antibiotics in Environmental Water Samples

Yuhao Wen [1,#], Mingyang Hou [1,2,#], Xingkai Hao [3], Dani Sun [1,2], Hao Zhang [1], Farooq Saqib [3], Wenhui Lu [4], Huitao Liu [2,*], Lingxin Chen [1] and Jinhua Li [1,2,*]

[1] Coastal Zone Ecological Environment Monitoring Technology and Equipment Shandong Engineering Research Center, Shandong Key Laboratory of Coastal Environmental Processes, Shandong Research Center for Coastal Environmental Engineering and Technology, Yantai Institute of Coastal Zone Research, Chinese Academy of Sciences, Yantai 264003, China

[2] College of Chemistry and Chemical Engineering, Yantai University, Yantai 264005, China

[3] ZJU-Hangzhou Global Scientific and Technological Innovation Center, Zhejiang University, Hangzhou 311215, China

[4] Faculty of Light Industry, Qilu University of Technology (Shandong Academy of Sciences), Jinan 250353, China

* Correspondence: liuht-ytu@163.com (H.L.); jhli@yic.ac.cn (J.L.)

# These authors contributed equally to this work.

Analysis of adsorption data model fitting

(1) Isothermal adsorption data model fitting

To conduct a comprehensive analysis of the isothermal adsorption characteristics of the MIPs, we employed two common thermodynamic models for evaluation: the Langmuir model and the Freundlich model [1-3]. The expression for the Langmuir model is presented in **Equation S1**:

$$Q_e = \frac{K_L Q_{max} C_e}{1 + K_L C_e} \tag{S1}$$

where Q_e (mg/g) is the equilibrium adsorption capacity of MIPs or MIPs to the target at different concentrations, and C_e (mg/L) is the initial concentration of the target in the solution. Q_{max} (mg/g) is the maximum adsorption capacity of MIPs to the target based on the core-shell monolayer under the premise that the imprinting site is fully utilized. K_L (L/mg) is the equilibrium constant of the Langmuir adsorption model.

(2) Dynamic adsorption data fitting

In order to explore the dynamic adsorption process and key parameters of MIPs, three classical kinetic models were used in this study: quasi-first-order kinetics, quasi-second-order kinetics, and intraparticulate diffusion models. These models are used to elucidate the behavior of the adsorbent during adsorption and to further predict the migration and distribution of target molecules within the adsorbent.

The expression of the quasi-first-order kinetic equation is usually shown in Equation S2:

$$Q_t = Q_e \left(1 - e^{-k_1 t}\right) \quad (S2)$$

where Q_t (mg/g) is the instantaneous adsorption capacity after the adsorption process at t time; Q_e (mg/g) is the maximum theoretical equilibrium adsorption capacity; k_1 is the adsorption rate constant of the quasi-first-order kinetic model.

The expression of the quasi-second-order kinetic equation is usually shown in Equation S3:

$$Q_t = \frac{Q_e^2 k_2 t}{1 + Q_e k_2 t} \quad (S3)$$

The definitions of Q_t(mg/g) and Q_e(mg/g) are consistent with the parameters in the quasi-first-order kinetic equation, and k_2 is the adsorption rate constant of the quasi-second-order kinetic model.

The intraparticulate diffusion model expression is typically shown in Equation S4:

$$Q_t = k_{id} t^{\frac{1}{2}} + c \quad (S4)$$

where Q_t (mg/g) represents the instantaneous adsorption capacity; K_{id} is the diffusion rate constant within the material; C is the model constant, which

can reflect the thickness of the interfacial layers

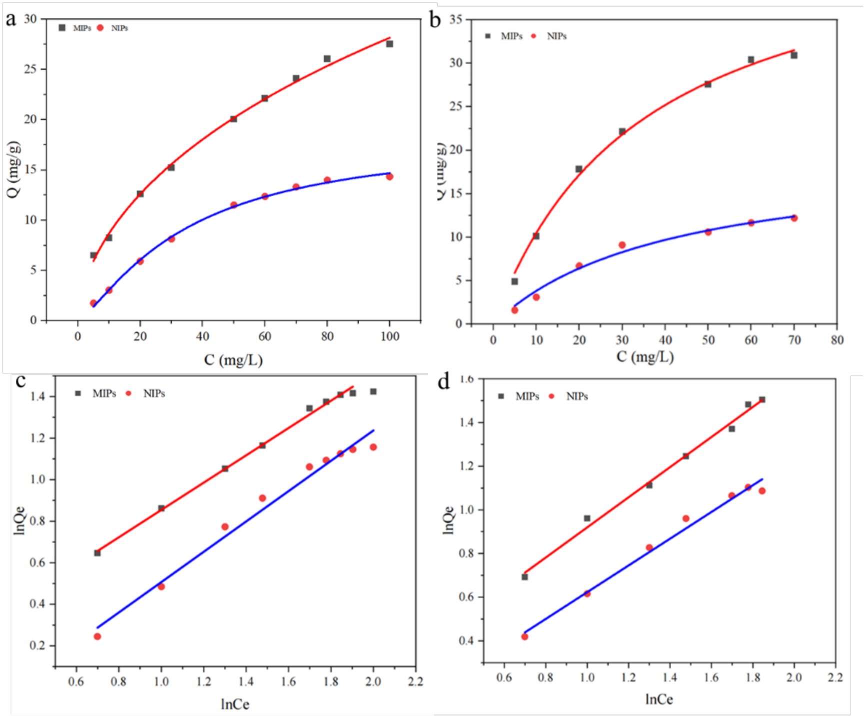

Figure S1. Langmuir adsorption curve fitting of (a) SMZ and (b) SMM; Freundlich adsorption curve fitting of (c) SMZ and (d) SMM.

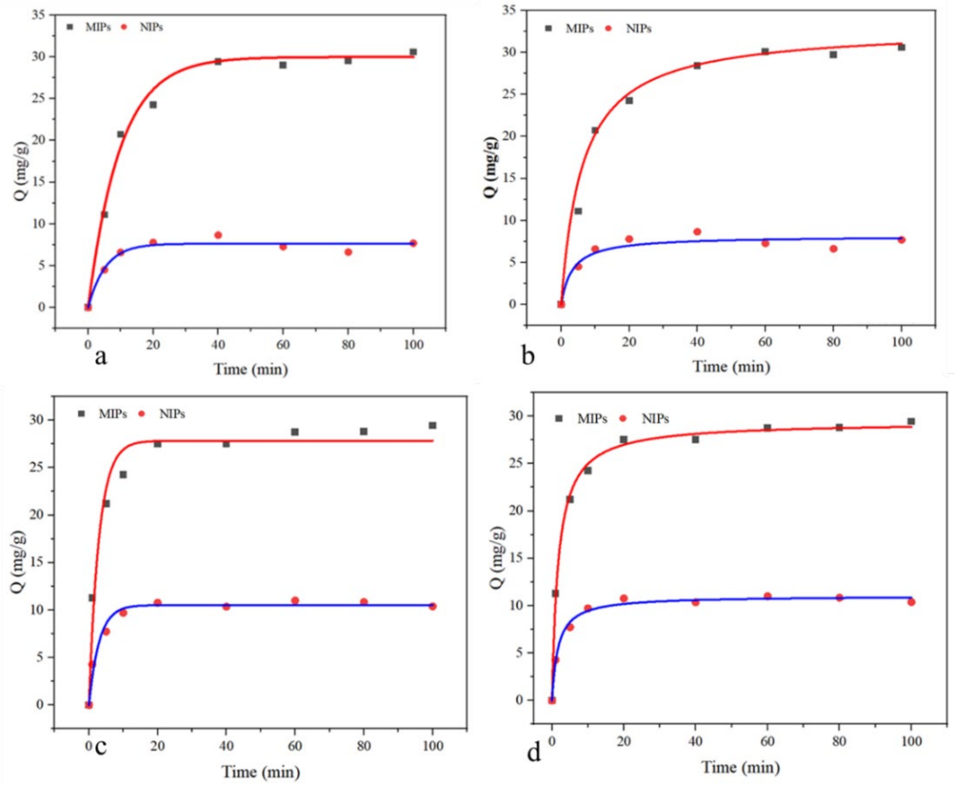

Figure S2. (a) and (b) fitting the SMZ pseudo first and pseudo second order kinetic adsorption curves; (c) and (d) fitting the SMM pseudo first and pseudo

second order kinetic adsorption curves.

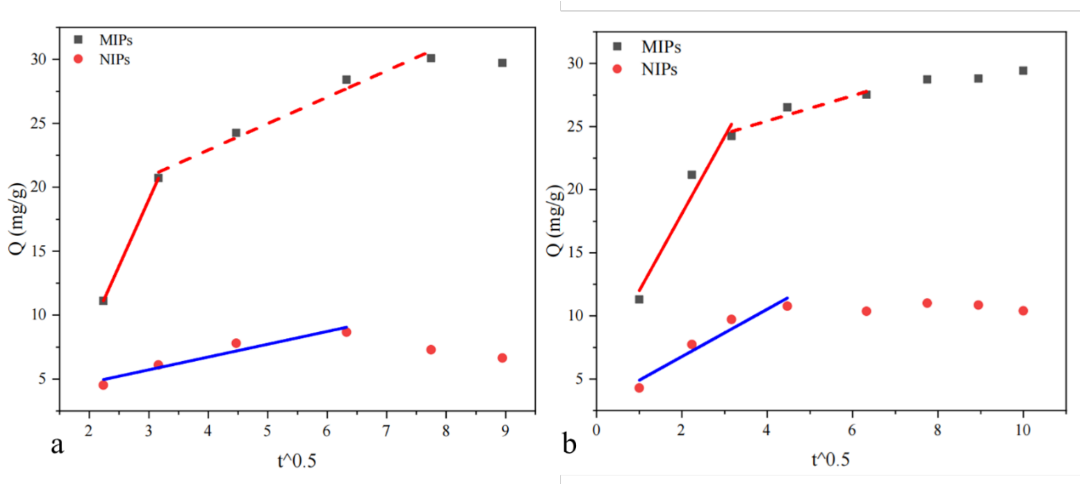

Figure S3. Internal adsorption curves of (a) SMZ and (b) SMM.

Table S1. Langmuir and Freundlich adsorption fitting data of dt-MIPs and NIPs.

Model	Parameter	SMZ		SMM	
		MIPs	NIPs	MIPs	NIPs
Langmuir	R^2	0.993	0.997	0.994	0.992
	Q_{max} (mg/g)	37.4±1.38	18.5±1.07	40.3±2.02	19.7±2.00
Freundlich	R^2	0.996	0.980	0.996	0.986
	1/n	0.66±0.016	0.73±0.038	0.61±0.033	0.79±0.026

Table S2. Three kinetic models fitting of dt-MIPs and NIPs.

Dynamical model	Parameter	SMZ		SMM	
		MIPs	NIPs	MIPs	NIPs

Pseudo-first-order	R^2	0.965	0.988	0.965	0.974
	Q_{max} (mg/g)	32.98±0.77	8.2±0.18	27.8±0.79	10.5±0.27
	K_1	0.0049±0.00132	0.038±0.0063	0.034±0.062	0.34±0.055
Pseudo-second-order	R^2	0.989	0.965	0.995	0.991
	Q_{max} (mg/g)	32.9±1.53	8.2±0.18	29.4±0.028	11.1±0.18
	K_2	0.005±0.0013	0.038±0.0063	0.02±0.002	0.05±0.008
Intra particle diffusion	R^2	1	0.927	0.955	0.932
		0.979	-	0.902	-
	K_{id}	10.36±1.230	2.72±0.856	6.11±1.317	1.88±0.357
		2.06±1.596	-	1.00±0.331	-
	C	-12.08±1.413	2.7±0.856	5.89±3.042	3.02±0.357
		14.64±1.231	-	21.44±1.597	-

Table S3. Analytical performance comparison of the dt-MIPs-DSPE-HPLC with reported SPE based methods for SAs determination.

Analyte	Method	Adsorbent	LOD	Samples	Ref.
SAs	Online MISPE-HPLC	Functionalized Silica Gel Sorbent	4.6, 5.1, and 7.3 ng L^{-1}	Pork and chicken muscle	[4]
SAs	OPD/MSPE-LC-FLD	Fe_3O_4/MWCNTs-OH	0.004–0.04 ng/g	Honey	[5]
SAs	SPE-HPLC-DAD	PEGDA polymer	7.5 and 16.2 μg kg^{-1}	Pork bacon, pork liver and chicken muscle	[6]
SAs	MSPE-DLLME-UHPLC-MS/MS	MWCNTs	1.02–2.97 ng L^{-1}	River water, lake water, influent wastewater, effluent wastewater and farm wastewater	[7]
SAs	DSPE-HPLC	MPS@SiO_2@MIPs	0.23–1.74 μg L^{-1}	Seawater, lakewater and tap water	This work

OPD/MSPE-LC-FLD: one-pot derivatization /magnetic solid-phase extraction- liquid chromatography-fluorescence detection;

PEGDA: Poly (ethylene glycol) diacrylate; MWCNTs: Multi-walled carbon nanotubes; MSPE-DLLME-UHPLC-MS/MS: Magnetic solid-phase extraction combined with dispersive liquid–liquid microextraction- ultra-high performance liquid chromatography-tandem mass spectrometry

References

1. Ezzati, R.A. new insight into the surface adsorption in the solution phase: A modification of the Langmuir isotherm, *Water Environ. Res.*, 2024, 96, e11019.

2. Pan, J.M.; Yao, H.; Guan, W.; Ou, H.X.; Huo, P.W.; Wang, X.; Zou, X.H.; Li, C.X. Selective adsorption of 2,6-dichlorophenol by surface imprinted polymers using polyaniline/silica gel composites as functional support: Equilibrium, kinetics, thermodynamics modeling, *Chem. Eng. J.*, 2011, 172, 847-855.

3. Basar, C.A. Applicability of the various adsorption models of three dyes adsorption onto activated carbon prepared waste apricot, *J. Hazard. Mater.*, 2006, 135, 232-241.

4. He, J.X.; Wang, S.; Fang, G.Z.; Zhu, H.P.; Zhang, Y. Molecularly imprinted polymer online solid-phase extraction coupled with high-performance liquid chromatography-UV for the determination of three sulfonamides in pork and chicken, *J. Agric. Food Chem.*, 2008, 56, 2919-2925.

5. Shi, N.; Liu, Y.W.; Li, W.X.; Yan, S.M.; Ma, L.; Xu, X.; Chen, D. One-pot derivatization/magnetic solid-phase extraction coupled with liquid chromatography-fluorescence detection for the rapid determination of sulfonamide residues in honey, *Food Chem.: X*, 2024,21, 101090.

6. Moga, A.; Vergara-Barberán, M.; Lerma-García, M.J.; Herrero-Martínez, J.M.; Simó-Alfonso, E.F.; Poly(ethylene glycol) diacrylate-based solid-phase extraction for determination of sulfonamides in meat samples, *Microchem. J.*, 2020, 157, 104931.

7. Yuan, X.C.; Wu, D.; Liu, C.; Li, X.H.; Xiong, Z.L.; Zhao, L.S. Polypyrrole-modified magnetic multi-walled carbon nanotube-based magnetic solid-phase extraction combined with dispersive liquid–liquid microextraction followed by UHPLC-MS/MS for the analysis of sulfonamides in environmental water samples, *New J. Chem.*, 2018, 42 (24), 19578-19590.
